# Analysing the association between perceived knowledge, and attitudes on Lassa Fever infections and mortality risk factors in lower Bambara Chiefdom

**DOI:** 10.1186/s12889-024-19170-w

**Published:** 2024-06-24

**Authors:** Abu-Bakarr S. Kamara, Andrew Moseray, Patrick Fatoma, Alhassan Mayei, Joseph Lamin, Osman Sankoh, Mohamed Kemoh Rogers

**Affiliations:** 1 Department of Environmental Health Sciences, School of Community Health Sciences, Njala University, Bo Campus, Bo City, Sierra Leone; 2 University of Management and Technology, Kissy Dockyard, Freetown, Sierra Leone; 3 School of Community Health Sciences, Bo Campus, Njala University, Sierra Leone Bo City,; 4 School of Public Health, Faculty of Health Sciences, University of the Witwatersrand, Johannesburg, South Africa

**Keywords:** Lassa fever, Knowledge, Attitudes, Prevention, Sierra Leone, Public health education

## Abstract

**Background:**

Lassa fever (LF) presents significant public health challenges in Sierra Leone, particularly in the Lower Bambara Chiefdom. This study aims to deeply understand how knowledge and attitudes towards LF correlate with community-driven prevention and control measures.

**Methods:**

A descriptive cross-sectional quantitative approach was used to conduct the research. Data from 2167 participants were collected using an Android-based survey from 1st February 2022 to 14th February 2022. Respondents' knowledge of LF causes, risk factors, transmission modes, and preventive measures were evaluated through a multiple-choice questionnaire, and attitudes toward prevention and control were measured on a 5-point Likert scale. Quantitative data were analyzed using SPSS version 26.0 and frequencies were presented in count, percentage, and table. Chi-square statistics were used to test for associations.

**Results:**

Among the 2167 participants, over half were males (1184, 54.60%), farmers (1406, 64.90%), married (monogamous) (1428, 65.90%), and had never attended school (1336, 61.70%). Respondents demonstrated high knowledge levels of LF across socio-demographic groups (33% to 100%) and shared a positive attitude towards prevention and control (mean score of 26.77 on a 5–40 scale). Educational level, religious beliefs, and occupational status significantly influenced LF knowledge (p < 0.05). Specifically, illiterates had a high knowledge score of 48.24%, while those with tertiary education had the highest score at 83.33%. Additionally, a Pearson correlation analysis revealed a positive linear relationship between the degree of knowledge and positive attitude towards LF infection and mortality risk factors (r = 0.090, p = 0.02).

**Conclusion:**

High LF knowledge in Lower Bambara Chiefdom positively influences prevention attitudes. Education, religion, and occupation are key factors. Tailored interventions enhance public health efforts.

**Supplementary Information:**

The online version contains supplementary material available at 10.1186/s12889-024-19170-w.

## Introduction

Lassa Fever (LF), a viral infectious illness prevalent in Sub-Saharan Africa, has caused a significant number of deaths in Sierra Leone, particularly in Tongo Field and Panguma in the Lower Bambara Chiefdom of the Kenema District. Ghana, Benin, and Mali have reported similar instances of LF morbidity and mortality [1]. The disease was initially discovered in the 1950s in West Africa, with the Lassa virus discovered in 1969 [2]. In Lassa, a small town in the Nigerian state of Niger, three American nurses got sick with the disease in 1969 [3]. Every year, the illness causes 300,000 cases of morbidity and 5,000 deaths. The World Health Organisation (WHO) estimates that the case fatality rate for LF is 1%, with a much higher risk among hospitalized patients with wet symptoms [3] [4],. The Mastomys rodent, which is prevalent in sub-Saharan Africa, has been related to several human pathogens [5] [6],. LF, a zoonotic illness, can be transmitted to humans via an infectious source that includes infected rodent tissue, blood, and excreta. In the majority of endemic regions, women of reproductive age have suffered greatly, contributing to a higher death rate than men [7]. Throughout the majority of Sierra Leone, LF is prevalent.

LF Case Fatality Rate (CFR) in Sierra Leone was thought to be 70% [8] but was assessed to be 67% and 63% among hospitalized patients and children [8] [7], respectively. The disease is more prevalent in rural areas, where living standards are lower and the probability of coming into contact with infected rodents is considerably high. Since Mastomys species rats are not native to Europe, there is no risk of getting the Lassa virus in a public place in the EU/EEA [7]. In the sub-Saharan region, particularly Nigeria, numerous studies on the knowledge, attitudes, and practices of LF have been conducted [1]. These ranged from an assessment of LF knowledge amongst health workers (physicians and nurses) in rural communities in the south-south zone of Nigeria, including Edo State [9, 10] to LF awareness amongst rural community dwellers in the southwest [11], an LF epicenter in Nigeria with several reported outbreaks, including the recent 2015/2016 epidemic [4]. Studies on community perception, knowledge, and practice on LF targeting community members based on sections have not been documented in Sierra Leone, whereas knowledge, attitude, and practice (KAP) studies have been documented in Nigeria focusing primarily on health care providers on how to prevent secondary transmission of the virus [12].

Knowledge is a powerful tool by used individuals and communities to make informed decisions about their health [13]. When people are aware of potential health risks, they are better equipped to take proactive steps to safeguard their health and well-being [13]. People who are informed about their health are more likely to engage in preventive behaviors [14]. For instance, during a health campaign, understanding whether people have concerns or reservations about avoiding social gatherings, performing burial rituals, eating bush meats, practicing social norms, etc. enables the creation of messages that specifically address their concerns. Public health initiatives become more successful, and individuals are more likely to follow recommended health measures when interventions are tailored to match the community's knowledge and attitudes which in turn improves community health as a whole [8]. Other underlying problems such as lack of education, and unwillingness of rural dwellers to adhere to public health messages, and traditional beliefs and practices further worsen the health situation of rural people [15]. Improper waste disposal results in breeding places for vectors of disease, while poor conditions housing offers easy access to rodents, which is common in rural homes [15]. These problems result in a significant spread of communicable diseases like LF thus adding to the local disease burden.

People’s knowledge about health risks is known to enhance appropriate preventive behaviors that will safeguard their health and the health of their communities [16]. Tailoring public health messages to align with the cultural norms and values of disease-prone and endemic communities increases the likelihood of acceptance and adherence within a community [17]. In this study, we evaluated the community's knowledge and attitudes regarding mortality-related risk factors for LF. Specifically, we aimed to elucidate how their level of knowledge influences their attitudes towards these risk factors.

## Materials and Methods

### Study design, setting, population, and sampling

A descriptive, cross-sectional quantitative design was employed for this study from 1st February 2022 to 14th February 2022. Descriptive studies are known to provide a better understanding of predisposing factors of disease conditions, whose occurrence could be related to individual and community attitudes and knowledge. In the context of this research, the design was crucial in providing a snapshot of the influence of community knowledge on their attitudes as it relates to the risk factors of LF at a specific point in time.

The study was conducted in Lower Bambara chiefdom, the LF endemic chiefdom of Kenema district in the eastern part of Sierra Leone [18]. The study targeted communities in all the six administrative sections known to be the Lassa belt with Tongo and Panguma as major towns both of which hosted the majority of the chiefdom population. These sections have a total population of 76,281 [19] which constitute the most Lassa-prone communities with the majority of all reported Lassa morbidity and mortality in Sierra Leone since it was discovered in the country in 1957. The chiefdom is inhabited by diverse ethnic groups and religious beliefs with the Mende ethnic group and Muslims predominating the area respectively [19].

Mining and farming are two of the most important economic activities providing livelihoods for locals in the study area. The Panguma Government Hospital, the Community Health Centre (CHC) in Tongo, and a network of smaller Peripheral Health Units (PHUs) provide health services within the chiefdom with the Panguma Catholic Hospital being the only health facility that diagnoses and refers Lassa patients to Kenema Government Hospital, the only specialized Lassa diagnosis and treatment hospital in Sierra Leone and the Mano River Union with a biosafety level 3 laboratory for diagnosis of viral hemorrhagic fevers (VHFs).

The study focused on adult males and females aged 18 years and older in the Lower Bambara Chiefdom of the Kenema district. The participants were either heads of households or knowledgeable household members acting as representatives. According to Statistics Sierra Leone, the Lower Bambara Chiefdom comprises 112 enumeration areas (EAs), which set the maximum sample size. We calculated a minimum sample size of 26 EAs using an Android sample size calculator application (relief Calculator), assuming a 95% confidence interval and a 0.05 precision level. Each of the 112 EAs in the sample frame was numbered from 1 to 112. Using a computer-based random number generator, an online software, every fifth EA was selected after counting from 1 to 4. In the 26 chosen EAs, all households were surveyed, resulting in a total of 2,167 responses.

### Data collection and processing

Data were collected using a structured, pre-tested questionnaire developed by the researchers in Microsoft Word, carefully considering the variables of interest. This questionnaire was then programmed into an Excel file and uploaded to the Ona platform, an Open Data Kit (ODK) server, for electronic data collection. The pre-tested questionnaire was installed on smartphones (Android devices), enabling enumerators to conduct the survey electronically and offline.

The questionnaire gathered information on respondents' demographics, knowledge, and attitudes towards LF. To ensure the questionnaire's consistency and reliability, it was piloted in the Kowama community, Bo district, which has similar characteristics to the study area. Feedback from enumerators and authors during this pilot, conducted a week before the main study, helped confirm the questionnaire’s validity.

To further ensure reliability, we calculated Cronbach's alpha for the knowledge and attitude sections of the questionnaire. Cronbach's alpha is a measure of internal consistency, indicating how closely related a set of items are as a group [20]. For the knowledge section, we achieved a Cronbach's alpha of 0.82; for the attitude section, we achieved a Cronbach's alpha of 0.85. Both values are above the commonly accepted threshold of 0.70, indicating the high reliability of the questionnaire.

Given the high illiteracy rate among the studied population, enumerators played a crucial role in administering the questionnaire. Twenty enumerators were trained to read the questionnaire accurately. The training was held for a week to ensure that enumerators were well-prepared to engage with participants effectively. They were instructed to read the questions aloud and record the responses accurately. This approach ensured that all participants, regardless of their literacy level, could fully engage with the survey and provide their responses.

The data collection period was relatively short, lasting two weeks. The 20 enumerators distributed into 5 teams of 4 members collected the data over these two weeks. Each team was at least 5-EAs. This intensive timeframe was necessary to ensure the timely completion of data collection while maintaining high data quality. The coordinated efforts of the trained enumerators allowed us to efficiently reach a substantial number of participants within this period, ensuring comprehensive coverage and accurate data gathering. Each household head or representative was interviewed face-to-face by a trained enumerator.

### Inclusion/exclusion criteria

To participate in the study, individuals had to be adult males or females aged 18 years and older, either the head of the household or a household member with good knowledge of the household, residing in selected EAs within Lower Bambara Chiefdom, and willing to serve as representatives of the household head. Eligible participants were those who consented to participate after the study's purpose and procedures were explained. Those who did not meet the set criteria were considered ineligible to participate.

### Data analysis

Collected data were double-entered, and analyzed on a computer using Statistical Package of Social Science Students (SPSS) version 26.0 software. The heart of our study lies in the associations identified during data analysis. We computed composite knowledge and attitude scores and performed bivariate and multivariate analyses. The 10-point knowledge questions were scored as + 1 for a favorable, positive, or correct answer, and -1 for an unfavorable, negative, or incorrect answer. Aggregate scores of 5 and above were regarded as 'good' while scores amounting to less than five were regarded as 'poor'. The Chi-square test examined associations between categorical variables at a significance level of p < 0.05. Frequencies were presented as proportions, percentages, charts, and tables to ensure a comprehensive understanding of the data.

### Measuring respondent’s knowledge *of Lassa* fever

Multiple choice questions were used to assess participants' knowledge about the prevention, causes, signs and symptoms, risk factors, and methods of contracting LF. Five multiple-select questions targeted these categories, with a total of 38 answer choices (marks) spread across them. Participants were required to select each correct option (1 mark per correct option); thus, omitting an option indicated a knowledge gap.

The total number of selected options across all variables was calculated out of a possible 38 to measure overall knowledge of LF. This count was used to determine the knowledge level of each participant. Participants’ scores from each question were summed to obtain an overall knowledge score per individual, and these scores were further aggregated for each community within the Lower Bambara Chiefdom.

Community scores were categorized into low, moderate, and high knowledge levels. Additionally, participants’ degrees of knowledge were cross-tabulated by community/section to analyze counts and proportions reflecting community-level knowledge and attitudes. A Chi-Square test was conducted for each cross-tabulation to determine the statistical association between knowledge levels and attitudes.

### Measuring respondent’s attitude towards Lassa fever prevention and control

Eight questions, each measuring attitude on a 5-point Likert scale (Strongly agree = 5; Agree = 4; Neutral = 3; Disagree = 2; Strongly Disagree = 1) were used to measure the degree of positive attitude for each participant. Each question was supposed to be a positive attitude. Therefore, participants who answered strongly agree are the most knowledgeable while those who answered strongly disagree are the least knowledgeable. The score for each question was aggregated to obtain an overall score of attitudes for each participant. Participants ‘scores were aggregated to attain an overall altitude score for the community. Community aggregated scores were computed to derive a categorical scale (degree of attitude) with options of low, moderate, and high positive attitude.

## Results

### Socio-demographic distribution and association *of Lassa* Fever knowledge among study subjects

Table 1 describes how different demographic factors are related to LF knowledge levels. It presents the socio-demographic characteristics of respondents and their level of knowledge, analyzed using a chi-square test. The data is categorized by sex, age, education, marital status, occupational status, and religion.

**Table 1:** Respondents socio-demographic characteristics and chi-square test of socio-demographic

Demographic variables		Level of knowledge								N	%	*p*. Value	Phi & Cramer's Value
		**High**		**Moderate**		**Low**		**None**					
		**n**	**%**	**N**	**%**	**n**	**%**	**n**	**%**				
**Sex**	Male	593	50.08	186	15.71	118	9.97	287	24.24	1184	54.64	0.339	0.039
	Female	510	51.88	156	15.87	76	7.73	241	24.52	983	45.36		
**Age**	18–25	95	47.03	35	17.33	21	10.40	51	25.25	202	9.32	0.077	0.045
	26–33	222	47.44	83	1 7.74	37	7.91	126	26.82	468	21.60		
	34–41	328	55.78	78	13.27	52	8.84	130	22.11	588	27.13		
	42–49	242	50.84	77	16.1 8	40	8.40	117	24.58	476	21.97		
	> 49	216	49.88	69	15.94	44	10.16	104	24.02	433	19.98		
**Education**	Illiterate	671	48.24	206	14.81	111	7.88	403	28.87	1391	64.19	0.001	0.104
	Primary	139	50.18	54	19.49	29	10.47	55	19.86	277	12.78		
	Junior Secondary	141	55.51	44	17.32	24	9.45	45	17.72	254	11.72		
	Senior Secondary	129	60.00	35	16.28	28	13.02	23	10.70	215	9.92		
	Tertiary	20	83.33	1	4.17	2	8.33	1	4.17	24	1.11		
	Non-formal	3	50.00	2	33.33	0	0.00	1	16.67	6	0.28		
**Marital Status**	Married (monogamous)	744	52.1 0	234	16.39	124	8.68	326	22.83	1428	65.90	0.069	0.075
	Married (polygamous)	181	47.26	61	15.93	39	10.18	102	26.63	383	17.67		
	Cohabiting	19	38.78	8	16.33	4	8.16	18	36.73	49	2.26		
	Single/never married	70	54.69	16	12.60	15	11.72	27	21.09	128	5.91		
	Widow/Widower	64	47.76	15	11.19	8	5.97	47	35.07	134	6.18		
	Divorced	6	60.00	2	20.00	0	0.00	2	20.00	10	0.46		
	Separated	18	56.25	6	18.75	2	6.25	6	18.75	32	1.48		
	Others	1	33.33	0	0.00	2	66.67	0	0.00	3	0.14		
**Occupational Status**	Farmer	700	50.1 8	219	15.70	121	8.67	355	25.45	1395	64.37	0.033	0.084
	Okada riding	17	50.00	2	5.88	1	2.94	14	41.18	34	1.57		
	Others	51	55.43	18	19.57	9	9.78	14	15.22	92	4.25		
	Miner	60	46.51	24	18.60	12	9.30	33	25.58	129	5.95		
	Teacher	35	60.34	13	22.41	5	8.62	5	8.62	58	2.68		
	Driver	5	41.67	0	0.00	2	16.67	5	41.67	12	0.55		
	Business	1 83	51.99	50	14.20	29	8.24	90	25.57	352	16.24		
	Student	48	53.93	15	16.85	15	16.85	11	1236	89	4.11		
	Hunting	0	0.00	0	0.00	0	0.00	1	100.00	1	0.05		
	Palm wine tapper	1	100.00	0	0.00	0	0.00	0	0.00	1	0.05		
	Health worker	3	75.00	1	25.00	0	0.00	0	0.00	4	0.18		
**Religion**	Muslim	911	49.11	296	15.96	163	8.79	485	26.15	1855	85.60	0.001	0.039
	Christian	192	61.54	46	14.74	31	9.94	43	13.78	312	1 4.40		

*p*. Value > 0.05 is considered significant

*Abbreviations*: *N* Population mean, *n* Sample mean, *%* Percentage

Among the 2167 participants, over half were males (54.60%), farmers (64.90%), married (monogamous) (65.90%), and had never attended school (61.70%). The most dominant age group was 34–41 years (27.10%). (Table 1).

Knowledge-wise; for sex, it shows 593 males (50.08%) with high knowledge, 186 (15.71%) moderate, 118 (9.97%) low, and 287 (24.24%) with none. Females had 510 (51.88%) with high knowledge, 156 (15.87%) moderate, 76 (7.73%) low, and 241 (24.52%). In a nutshell, both males (50.08%) and females (51.88%) have similar knowledge distributions of LF and have no statistically significant significance with LF knowledge (*p* = 0.33) (Table 1). Moreover, age groups showed varying and weak trends of increasingly high knowledge levels. Those aged 34–41 years 328 (55.78%) and 42–49 years; 242 (50.84%) have the highest level of knowledge. The Chi-square (*p* = 0.077), suggest no significant difference across age groups. In addition, education levels varied significantly in knowledge, with illiterates at 671 (48.24%) and individuals that have attained tertiary education at 20 (83.33%) recording the highest LF knowledge. Educational level has a statistical significance with LF knowledge (*p* = 0.001) (Table 1).

Furthermore, marital status also showed differences in LF knowledge. Participants that were married (monogamous) 744 (52.10%) with high knowledge, while those cohabiting had 19 (38.78%) with high knowledge. The Chi-square (*p* = 0.069) also, suggests some significant differences in knowledge levels based on marital status. In occupational status, farmers had 700 (50.18%), and palm wine tappers 1 (100%) had the highest LF knowledge. The Chi-square (*p* = 0.033), indicated significant differences in knowledge levels based on occupation (Table 1).

Religiously, Muslims had 911 (49.11%) with high knowledge, as well as Christians had 192 (61.54%). Religion shared significant differences (*p* = 0.001) in LF knowledge levels (Table 1).

### Respondent's attitude towards Lassa Fever preventive measures

Table 2 presents a detailed analysis of respondents' attitudes towards various preventive measures for LF control across the sampled sections in Lower Bambara chiefdom. In terms of preventing rodent entry into homes, the chiefdom level exhibited a mean score of 3.19, with a standard error of 0.23 and a 95% confidence interval ranging from 3.15 to 3.24. Scores varied between a minimum of 2 and a maximum of 5. Notably, Bonya section reported the highest mean score (3.37), followed by Fallay (3.26) and Nyawa and Gboro (both 3.07), while Korjei Ngieya and Sei sections recorded the lowest scores (2.96 and 2.92, respectively). Similarly, attitudes towards thoroughly washing uncooked food before consumption were measured. The overall mean score at the chiefdom level was 3.29, with a standard error of 0.017 and a confidence interval between 3.26 to 3.33. All sections reported mean scores above 3.0, with Fallay having the highest mean score of 3.45, followed by Gboro (3.32) and Nyawa (3.30) (Table 2).

**Table 2:** Respondents’ attitude and overall measure of Lassa fever knowledge

Participants attitudes towards preventing rodents from entering homes							
Sections	**Frequency (N)**	**Mean**	**Std. Deviation**	**Std. Error**	**95% CI for Mean**	**Minimum**	**Maximum**
Bonya	467	3.43	1.073	0.050	(3.34, 3.53)	2	5
Fallay	47	3.23	1.183	0.173	(2.89, 3.58)	2	5
Gboro	78	3.08	1.029	0.117	(2.84, 3.31)	2	5
Korjei Ngieya	63	3.03	0.967	0.122	(2.79, 3.28)	2	5
Nyawa	514	3.10	1.066	0.047	(3.00, 3.19)	2	5
Sei	52	2.88	1.022	0.142	(2.60, 3.17)	2	5
Total	1221	3.22	1.077	0.031	(3.16, 3.28)	2	5
Attitude towards thoroughly washing uncooked food before eating							
Bonya	467	3.33	0.808	0.037	(3.25, 3.40)	2	5
Fallay	47	3.51	0.856	0.125	(3.26, 3.76)	2	5
Gboro	78	3.24	0.724	0.082	(3.08, 3.41)	2	5
Korjei Ngieya	63	3.24	0.560	0.071	(3.10, 3.38)	2	4
Nyawa	514	3.36	0.815	0.036	(3.29, 3.43)	2	5
Sei	52	3.15	0.638	0.088	(2.98, 3.33)	2	5
Total	1221	3.33	0.792	0.023	(3.29, 3.38)	2	5
Attitude towards properly covering all foods and drinking water							
Bonya	467	3.27	0.670	0.031	(3.20, 3.33)	2	5
Fallay	47	3.45	0.775	0.113	(3.22, 3.67)	2	5
Gboro	78	3.19	0.604	0.068	(3.06, 3.33)	2	5
Korjei Ngieya	63	3.05	0.418	0.053	(2.94, 3.15)	2	4
Nyawa	514	3.31	0.709	0.031	(3.25, 3.37)	2	5
Sei	52	3.13	0.561	0.078	(2.98, 3.29)	2	5
Total	1221	3.27	0.675	0.019	(3.23, 3.31)	2	
Attitude towards use of rodent poison as an effective way for controlling rodents							
Bonya	467	3.48	0.822	0.038	(3.40, 3.55)	2	5
Fallay	47	3.51	0.804	0.117	(3.27, 3.75)	2	5
Gboro	78	3.40	0.795	0.090	(3.22, 3.58)	2	5
Korjei Ngieya	63	3.32	0.758	0.096	(3.13, 3.51)	2	5
Nyawa	514	3.48	0.836	0.037	(3.41, 3.55)	2	5
Sei	52	3.48	0.874	0.121	(3.24, 3.72)	2	5
Total	1221	3.47	0.824	0.024	(3.42, 3.51)	2	5
Attitude towards properly covering and keeping cooking utensils							
Bonya	467	3.51	0.872	0.040	(3.43, 3.59)	2	5
Fallay	47	3.60	0.742	0.108	(3.38, 3.81)	2	5
Gboro	78	3.45	0.847	0.096	(3.26, 3.64)	2	5
Korjei Ngieya	63	3.43	0.712	0.090	(3.25, 3.61)	2	5
Nyawa	514	3.52	0.912	0.040	(3.44, 3.60)	2	5
Sei	52	3.31	0.805	0.112	(3.08, 3.53)	2	5
Total	1221	3.50	0.873	0.025	(3.45, 3.55)	2	5
Attitude towards discouraging storage of food in the roof							
Bonya	467	3.51	1.256	0.058	(3.39, 3.62)	1	5
Fallay	47	4.02	1.207	0.176	(3.67, 4.38)	1	5
Gboro	78	3.81	1.163	0.132	(3.55, 4.07)	1	5
Korjei Ngieya	63	3.75	1.204	0.152	(3.44, 4.05)	1	5
Nyawa	514	3.65	1.185	0.052	(3.55, 3.75)	1	5
Sei	52	3.71	1.143	0.159	(3.39, 4.03)	1	5
Total	1221	3.63	1.215	0.035	(3.56, 3.70)	1	5
Overall measure of positive attitudes towards lf infection and mortality risk factors							
Bonya	467	26.69	3.949	0.183	(26.33, 27.05)	19	40
Fallay	47	27.83	4.156	0.606	(26.61, 29.05)	20	37
Gboro	78	26.50	3.930	0.445	(25.61, 27.39)	19	37
Korjei Ngieya	63	25.63	2.484	0.313	(25.01, 26.26)	20	30
Nyawa	514	26.99	4.048	0.179	(26.64, 27.34)	19	40
Sei	52	26.06	2.859	0.396	(25.26, 26.85)	20	35
Total	1221	26.77	3.910	0.112	(26.55, 26.99)	19	40

*Abbreviations*: *Std. Deviation* Standard deviation, *Std. Error* standard error, *CI* Confidence interval

The study also assessed attitudes towards covering food and drinking water. The findings indicated a generally positive attitude across sections, with the mean scores consistently exceeding 3.0. Fallay achieved the highest mean score of 3.45, followed by Bonya with 3.27, whereas Korjei Ngieya had the lowest mean score of 3.05. In the context of using rodent poison as an effective control measure, the sectional means again surpassed the 3.0 threshold. The highest means were observed in Fallay (3.51), Nyawa and Bonya (both 3.48), and Sei and Gboro (3.40).

Furthermore, the survey evaluated attitudes toward covering and storing cooking utensils properly. The sections of Fallay, Nyawa, and Bonya showed the highest mean scores of 3.60, 3.52, and 3.51, respectively, followed by Gboro (3.45) and Korjei Ngieya (3.43) (Table 2). Regarding the discouragement of storing food on roofs, the sections of Fallay, Gboro, Korjei Ngieya, and Sei exhibited higher mean scores of 4.02, 3.81, 3.75, and 3.71, respectively. Additionally, Nyawa and Bonya also showed significant mean scores in this regard (Table 2).

### Respondents’ overall knowledge and attitude *of Lassa* Fever across lower Bambara sample

The study further compiled an overall measure of positive attitudes towards LF infection and mortality risk factors by summing the scores of eight questions. The chiefdom's mean score was 26.77 on a 5–40 scale, with a standard deviation of 3.910, a standard error of 0.112, and a confidence interval ranging from 26.55 to 26.99. Scores ranged from a minimum of 19 to a maximum of 40. Lastly, a Pearson Correlation Coefficient was computed to ascertain the linear relationship between the degree of knowledge and positive attitude toward LF infection and mortality risk factors among respondents with prior knowledge of LF (*N* = 1221). The analysis revealed a positive linear relationship between these variables (r (df) = 0.090, *p* = 0.02) (Table 3).

**Table 3:** Association between knowledge and attitude towards lf infection and mortality risk factors

		Degree of knowledge	Degree of positive attitude
Degree of knowledge	Pearson correlation	1	0.090^a^
	Sig. (2-tailed)		.002
	Sum of Squares and Cross-products	53,204.826	2827.344
	Covariance	43.611	2.317
	N	1221	1221
Degree of positive attitude	Pearson Correlation	0.090^a^	1
	Sig. (2-tailed)	.002	0
	Sum of Squares and Cross-products	2827.344	18,652.477
	Covariance	2.317	15.289
	N	1221	1221

^a^Correlation is significant at the 0.01 level (2-tailed)

## Discussions

LF is an acute viral hemorrhagic illness caused by the Lassa virus, belonging to the Arenaviridae family [21, 22]. Endemic to parts of West Africa, including Sierra Leone [14, 16, 23], and has significantly impacted the Lower Bambara Chiefdom in Kenema District, presenting a serious public health challenge for the local community [24, 25]. It was discovered that LF was present in this region during the study of an outbreak that occurred in the Lower Bambara chiefdom in December 2016 [18] and has the highest infection rate of the disease worldwide [24–26] attesting LF endemicity in Kenema District's Lower Bambara chiefdom.

The organization of domestic spaces brings together humans and rodents and creates pathways for infection in rural settlements [22]. Good knowledge of LF and adequate preventive measures for the disease could reduce the rate and spread of Lassa virus infection and a gap in knowledge of LF and its transmission mechanisms can therefore read communities susceptible to disease transmission. The high level of knowledge and positive attitude observed among participants can be attributed to the high prevalence of LF in the Lower Bambara Chiefdom. This region has historically experienced frequent outbreaks of LF, which has likely heightened community awareness and understanding of the disease. The continuous exposure to the risk and impact of LF has necessitated the dissemination of information and the adoption of preventive measures within the community consequently developing a substantial knowledge base and a proactive attitude towards controlling and preventing the disease.

According to the study, both males and females have similar levels of knowledge regarding LF. This finding is consistent with another research conducted in Africa. For example, a study in Liberia has frequently reported minimal gender differences in terms of awareness and understanding of LF [9]. From the current study, almost all of the study participants knew LF and its deadliness. These findings vary significantly by studies done in Nigeria which demonstrated fair knowledge but a gap in knowledge of the disease severity and mode of transmission [1] [20], [21], However, the current finding was in agreement with a knowledge assessment research done among rural communities of Ebonyi State Nigeria which showed a high level of LF disease understanding [27]. Similarly, older age groups in various African settings have been shown to possess more comprehensive knowledge about LF, likely due to their cumulative life experiences and prolonged exposure to health information [28]. This aligned with the present results which showcased a high level of knowledge among the elderly population. The present study noted that education has a significant impact on LF knowledge, which is a common observation across Africa [29, 30]. Several studies conducted across the continent have shown that individuals with higher educational qualifications have a better understanding of diseases and their management [17, 31]. This trend highlights the crucial role of education in improving health literacy among African populations. Furthermore, marital status has shown no significant impact on LF knowledge based on the current findings. This finding is somewhat different from studies conducted in other African regions, where marital status can be an important factor in health knowledge, especially in cultures where information is commonly shared within families [17, 32]. Occupational status was also reported to play a significant role in the knowledge of LF according to the recent result. This finding is consistent with another African research that suggests occupations with greater exposure to environmental risk factors associated with the disease tend to have higher awareness levels [7]. The impact of occupation on disease knowledge is a well-documented phenomenon in several African studies. Nevertheless, another Nigerian study showed no association between occupation and knowledge [33]. The significant difference in LF knowledge between Muslims and Christians in the current study highlights the role of religion in shaping health knowledge, a factor that can vary greatly across Africa. Religious beliefs and practices can significantly influence health behaviors and knowledge, though this influences LF [34].

Regarding the clinical manifestation of LF, the current study suggests that there was insufficient knowledge of LF among study participants in all study locations in Lower Bambara chiefdom as none of the six sections had mean values equal to 5.5 which is half of 11 (total marks used to assess knowledge) which indicates that the chiefdom does not have a good knowledge on LF clinical signs and symptoms although there is a fair knowledge across sections. This result was in contrast with a similar research done in Nigeria [1] which reported that study participants had a good knowledge about the precise clinical presentation of LF.

The study also identified that more than half of the participants were aware that LF could be contracted through direct contact with the blood and bodily fluids of infected individuals. This was consistent with a study conducted in Nigeria which reported that 68% of the respondents had appropriate knowledge of the means of contracting the viral disease through infected human bodily fluid and blood [35].

Based on sectional disparities, Bonya and Korjei Ngieya were the most knowledgeable sections on the methods of contacting LF and were with residents of Ebonyi State [27].

The overall knowledge about the causes of LF in the sampled sections is relatively low (the average score is much closer to 0 than to 7), with some variation in knowledge levels among the population. According to the study, Bonya and Gboro were the most knowledgeable sections on the causes of LF. The findings of this study were not consistent with results from a Nigerian study highlighting high-level knowledge on LF causes [1]. However, another research reported that cultural beliefs on the knowledge of LF among the respondents could not also be overemphasized [35].

The current study further suggests that more than half and one-third believe that LF is caused by the devil and can be used to afflict a person. Respondents’ perceptions of LF were in line with studies done in Ethiopia and Nigeria [35–37]. The current study suggests that there was adequate knowledge in preventing LF viral infection in Lower Bambara chiefdom. The findings of this study was collaborated by [7, 9] which reported that 93% of the respondents were informed about the preventive strategies for rats at home including the use of rodenticide, and storing of dried food in sealed containers.

The latest study indicates a moderate overall attitude towards LF prevention and control, with mean scores ranging from neutral to agree. This finding aligns with other studies that suggest attitude variations based on factors like disease prevalence, public health education, and cultural beliefs [38, 39]. Notably, the study also identified slight but noticeable variations in attitudes among individuals in different sections of the Lower Bambara Chiefdom. This variability echoes findings from other research, which often attributes such differences to demographic factors [40]. Besides, personal experiences with the disease, perceived severity, and trust in health authorities are key themes in related studies that influence attitudes toward LF prevention [6, 38]. Moreover, research emphasizes the importance of effective awareness campaigns in shifting attitudes from neutral to more positive and the significant role of public health interventions in this regard [41–44].

It is worth noting that the study has some limitations. One notable limitation is that the research was conducted in only 26 out of the 112 EAs in the Lower Bambara Chiefdom. This limited geographic scope means that the findings may not fully represent the entire population, as a significant portion of the community did not have the opportunity to participate in the study. Future research should aim to include a broader sample to enhance the generalizability of the results and provide a more holistic understanding of community knowledge and attitudes towards LF.

## Conclusion

Our study provides a comprehensive analysis of the knowledge and attitudes towards LF among residents of Lower Bambara Chiefdom. The findings underscore the necessity for targeted educational initiatives to enhance understanding of LF's signs, symptoms, causes, and transmission methods. While attitudes towards LF preventive measures were generally positive, there is a need to further deepen awareness, raise community sensitization, and implement tailored interventions targeting sub-groups in the population in Lassa endemic areas. These efforts are essential to meet the study's objective of improving community-driven prevention and control measures for LF.

### Supplementary Information

Supplementary Material 1.

## Data Availability

Data is provided in the manuscript presented in tables. However, there is privacy regarding the raw data and it can only be made available based on request and upon permission from the corresponding authors.

## Abbreviations

LF: Lassa fever
EA: Enumeration Area
VHF: Viral Hemorrhagic Fever
