# Supplementary material for: Analysing the association between perceived knowledge, and attitudes on Lassa Fever infections and mortality risk factors in lower Bambara Chiefdom
Source: BMC Public Health. 2024 Jun 24;24:1684. doi: 10.1186/s12889-024-19170-w (PMC11197251; doi:10.1186/s12889-024-19170-w)
Supplement: Supplementary file 1 — Supplementary Material 1. [file 12889_2024_19170_MOESM1_ESM.docx]

**QUESTIONNAIRE**

NJALA UNIVERSITY

SCHOOL OF COMMUNITY HEALTH SCIENCES

DEPARTMENT OF ENVIRONMENTAL HEALTH SCIENCE

**PhD Research in Public Health**

***A Study to Assess the burden of Lassa fever Infection and Mortality in Sierra Leone***

Hello. My name is…………………………………………………………………………………………… I am from Njala University, Department of Environmental Health Sciences, School of Community Health Sciences. We are conducting a study to estimate Lassa fever infection and deaths in Sierra Leone. In this study, we will discuss with selected members of the household to find out their perceptions and knowledge about the risk factors influencing Lassa fever infection and deaths. Also, the study will try to determine deaths in your community that are Lassa fever-related. The interview will be conducted in three phases at the household level. The information we collect will help the Government and Partners to estimate Lassa Fever infection and deaths especially those deaths that occur in communities that are not recorded by the conventional health care system and what they should do further to improve on the prevention, control and treatment of Lassa Fever in the Country. All of the information you share with us will be kept confidential – meaning that we will not link your name in any reports or identify you as the respondent in any other way. Your participation in this study is voluntary, and therefore you will not be paid. The discussion will take about ***45 – 60 minutes.*** You may decide to stop the interview at any point or refuse to answer questions you do not feel comfortable responding to. There are no right or wrong responses. Therefore, we encourage you to be honest and truthful in your responses so that we can accurately understand the situation on the ground.

Do I have your permission to continue with the interview?

**PART ONE: SAMPLE HOUSEHOLD ENUMERATION**

Enumerator ID………………………………

Date of interview……………………../…………………./2021

(Day) (Month)

Name of Location…………………………….. Household No…………………………

Name of District………………………………. Name of Chiefdom……………………

Name of Section……………………………… EA code………………………………

Record GPS coordinate of the community………………………

| **A-1** Name of Respondent | ( ) |
| --- | --- |
| **A-2** Sex of respondent | 1. Male 2. Female 3. Other |
| **A-3** Age of Respondent | ˂18 **Skip to QA-5**  ˂6 yrs. **Skip to** **SECTION B** |
| **A-4**. Relationship of the respondent to the household head | 1. Head 2. Spouse 3. Parent 4. Son/daughter 5. Brother or Sister 6. others Specify ( ) |
| A-5 Is he/she attending school? | Yes  No If No to QA-5 skip to QA-7 |
| **A-6** Educational status of respondent | 1. Illiterate   NOTE; This Q only appears if subject is 6 yrs. & above   1. Primary 2. Junior secondary 3. Senior Secondary 4. Tertiary 5. Others Specify ( ) |
| **A-7** Marital status of respondent | 1. Married (Monogamous) 2. Married (polygamous) 3. Cohabiting 4. Single/never married) 5. Widow/Widower 6. Divorced 7. Separated |
| **A-8** What is his/her religion? | 1. Muslim 2. Christian 3. Other, Specify ( ) |
| **A-9** Occupational status of respondent | 1. Farmer 2. Miner 3. Teacher 4. Driver 5. Business 6. Student 7. Hunting 8. Palm wine tapper 9. Health worker 10. Okada riding 11. Others, Specify ( ) |

**Section A: Personal Information of the respondent**

**Section B: Information of the Household**

**PART TWO: COMMUNITY CHARACTERISTICS AND PERCEIVED KNOWLEDGE AND ATTITUDE**

**ON LASSA FEVER INFECTION AND MORTALITY RISK-RELATED FACTORS**

**Section C: Information on community perceived knowledge and attitude towards LF**

| **C-1** Have you got any knowledge about Lassa fever? | 1. Yes (skip) 2. No |
| --- | --- |
| C-2 What source did you learn about Lassa fever?  **(Please select as many as applicable)** | 1. Friend 2. Family 3. Massa media 4. Hospital 5. Religious leaders 6. School 7. Others, please specify ( ) |
| C-3 State the signs and symptoms of Lassa fever    **(Please select as many as applicable)** | 1. Fever 2. Headache 3. Chest pain 4. Sore throat 5. vomiting 6. Diarrhoea 7. Bleeding from the orifices 8. Redness of the eyes 9. Cough 10. Vomiting 11. Facial swelling 12. Abortion of pregnancy 13. Others, please specify ( ) |
| C-4 How can one contract Lassa fever?  **(Please select as many as applicable)** | 1. Eating rats 2. Contacting infected rodent fluid/feaces 3. Contacting Lassa Infected person 4. Pricked with infected needles and sharps 5. Blood transfusion 6. Rat infested food 7. Others, please specify ( ) |
| C-5 what are the causes of Lassa fever?  **(Please select as many as applicable)** | 1. Unhygienic food practices 2. Open refuse disposal 3. Poor hand hygiene 4. Farming practices 5. Mining activities 6. Charcoal burning 7. Poor food storage practices 8. Others, please specify ( ) |
| C-6 What is/are the risk(s) of getting Lassa fever? | 1. Living in rural communities 2. Close contact with sick persons 3. Uncovered stored food 4. Improper waste management and dirty environment 5. Spreading food items on ground 6. Palm-wine consumption 7. Drinking water from open source(s) |
| C-6 How can Lassa fever be prevented? | 1. Hygienic food practices 2. Proper waste management 3. Proper hand hygiene 4. Good farming practices 5. Good mining activities 6. Avoid charcoal burning 7. Proper food storage practices 8. Others, please specify……………………………………… |

**C-7**

| **VARIABLE** | **Agree** | **Strongly agree** | **Neutral** | **Disagree** | **Strongly disagree** |
| --- | --- | --- | --- | --- | --- |
| Rodents should be prevented from entering the home |  |  |  |  |  |
| Uncooked food should be washed thoroughly before eating |  |  |  |  |  |
| All foods and drinking water should be properly covered |  |  |  |  |  |
| Use of rodent poison is effective for controlling rodents |  |  |  |  |  |
| All rodents should be killed at sight in the home |  |  |  |  |  |
| All toiletries should be kept in closed containers |  |  |  |  |  |
| Cooking utensils and cutleries should be properly covered and kept |  |  |  |  |  |
| Storage of food in the roof should be discouraged |  |  |  |  |  |
